# Protocols for protein-DNA binding analysis of a zinc finger transcription factor bound to its cognate promoter

**DOI:** 10.1016/j.xpro.2022.101598

**Published:** 2022-07-31

**Authors:** Lana C. Ly, Yang Yang, Fudong Li, Merlin Crossley, Yunyu Shi, Kate G.R. Quinlan

**Affiliations:** 1 School of Biotechnology and Biomolecular Sciences, University of New South Wales, Sydney, NSW 2052, Australia; 2 School of Life Sciences, Division of Life Sciences and Medicine, University of Science and Technology of China, Hefei, Anhui 230026, China; 3 Ministry of Education Key Laboratory for Membraneless Organelles and Cellular Dynamics, University of Science and Technology of China, Hefei, China

**Keywords:** Biophysics, Molecular Biology, Protein Biochemistry, Protein expression and purification, X-ray Crystallography

## Abstract

Here, we describe protocols to interrogate the binding of the zinc fingers of the transcription factor ZBTB7A to the fetal *γ-globin* (*HBG*) promoter. We detail the steps for performing electrophoretic mobility shift assays (EMSAs), X-ray crystallography, and isothermal titration calorimetry (ITC) to explore this interaction. These techniques could readily be applied to the structural studies of other zinc finger transcription factors and cognate DNA sequences.

For complete details on the use and execution of this protocol, please refer to Yang et al. (2021).

## Highlights

• Steps described for electrophoretic mobility shift assays (EMSAs)
• X-ray crystallography and isothermal titration calorimetry (ITC)
• Interrogate ZBTB7A interaction with fetal globin promoter

## Abstract (editor highlights)

Publisher’s note: Undertaking any experimental protocol requires adherence to local institutional guidelines for laboratory safety and ethics.

## Graphical abstract

**Figure:**
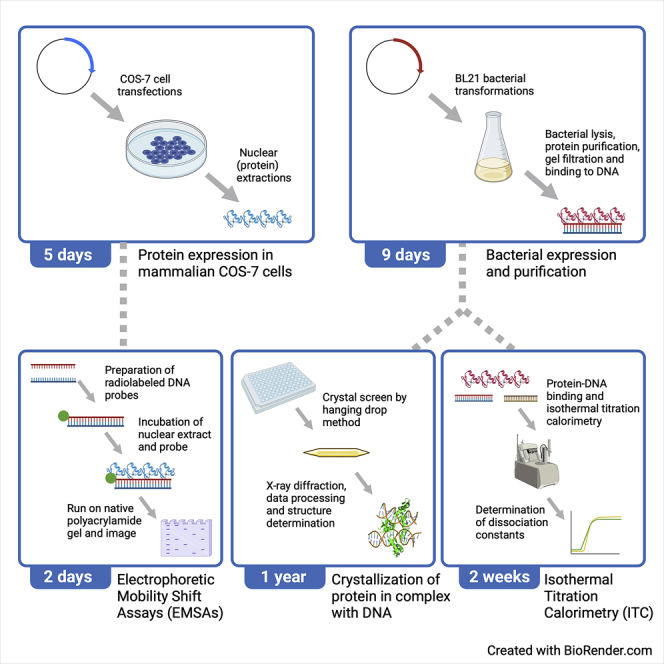

## Before you begin

Eukaryotic cells can be regulated by transcription factors that activate or repress gene expression. Many of these transcription factors contain small protein structural motifs known as zinc fingers that function as DNA binding domains. The functional versatility of zinc finger transcription factors is due to the amino acid residues within each zinc finger that allow for recognition of specific DNA sequences. Knowledge of transcription factor binding sites allows us to predict target genes. *In vitro* methods such as DNA-protein binding assays and X-ray crystallography can help to determine the precise DNA motif zinc fingers can bind to at a specific genomic region.

The protocols below describe the detailed steps for structural and biochemical methods for studying the structure of ZBTB7A bound to the γ-*globin* -200 element (Yang et al., 2021). We first describe the expression of zinc finger (ZF) DNA-binding domains of ZBTB7A in mammalian cells and electrophoretic mobility shift assays (EMSAs) performed to explore protein-DNA binding *in vitro*. Next, we describe the procedures for the bacterial expression and purification of the C-terminal ZF DNA-binding domain of ZBTB7A. We then describe the procedures for crystallization of ZBTB7A in complex with -200 element. Lastly, we describe the isothermal titration calorimetry (ITC) method to determine precisely how patient DNA mutations interfere with ZBTB7A binding.

The protocol can be adapted to examine other eukaryotic and prokaryotic transcription factors and their interactions with target DNA sequences.

### Institutional permissions

The work conducted at UNSW Sydney was approved by the UNSW Gene Technology Research Committee (GTRC) (approval: NLRD 17-09) and the UNSW Radiation Safety Committee (reference number: RSC 13-2021). It is important to acquire permissions from the relevant institutions.

## Key resources table

**Table:** 

REAGENT or RESOURCE	SOURCE	IDENTIFIER
**Bacterial and virus strains**		

*E. coli* BL21(DE3) competent cells	Novagen	N/A

**Chemicals, peptides, and recombinant proteins**		

ZBTB7A (amino acids 382-506)	This paper	N/A
ATP, [γ-^32^P] (250 μCi)	PerkinElmer	Cat# BLU502A250UC
Ampicillin	Solarbio	Cat# A8180-5G
IPTG	BioFroxx	Cat# 1122GR100
TCEP	Sigma	Cat# C4706-2G
cOmplete ULTRA Tablets, Mini, EDTA-free EASYpack	Roche	Cat# 05892791001
L-Glutathione (Reduced)	AMRESCO	Cat# 0399-50G
HEPES	Thermo Fisher Scientific	Cat# 15630106
Tris	Solarbio	Cat# T8060-500G
EDTA	Sigma	Cat# E6635
Boric acid	Sigma	Cat# B0394
NaCl	Sigma	Cat# 71379
CaCl_2_	Sigma	Cat# 21098
KCl	Sigma	Cat# 746436
MgCl_2_	Sigma	Cat# M8266
MgSO_4_	Sigma	Cat# 63138
ZnSO_4_	Sigma	Cat# 96500
KH_2_PO_4_	Sangon	Cat# A100781-0500
NaOH	Sangon	Cat# A100583-0500
HCl	Sinopharm Chemical Reagent	Cat# 10011018
NH_4_Cl	Sangon	Cat# A100621-0500
D-(+)-Glucose	Sangon	Cat# A100188-0500
TEMED	Sangon	Cat# A610508-0100
Ammonium persulfate (APS)	Sangon	Cat# A100486-0100
Poly(deoxyinosinic-deoxycytidylic) acid sodium salt (poly dIdC)	Sigma	Cat# P4929
SDS	Sangon	Cat# A100227-0500
10× Tris-Tricine	Sangon	Cat# C506039-0500
Acryl/Bis 40% Solution (19:1)	Sangon	Cat# B546012-0500
Tryptone	Oxoid	Cat# LP0042B
Yeast extract	Oxoid	Cat# LP0021
Agarose	Spanish	Cat# 111860
D2000 plus DNA ladder	Solarbio	Cat# M1070-100T
Unstained Protein MW Marker	MBI	Cat# SM0431
PrimeSTAR Max DNA Polymerase	Takara	Cat# R405B
*Nde*I	Takara	Cat# 1161A
*Xho*I	Takara	Cat# 1094A
DNA Ligation Kit (Version 2.1)	Takara	Cat# 6022
DNase I, RNase-free (1 U/μL)	Thermo Fisher Scientific	Cat# EN0521

**Critical commercial assays**		

Quick Spin Columns for radiolabeled DNA Purification Kit (Sephadex G-25 columns)	Sigma	Cat# G25DNA-RO
AxyPrep Plasmid Miniprep Kit (Nucleic Acid Purification Kit)	Axygen	Cat# AP-MN-P-250
E.Z.N.A. Gel Extraction Kit	Omega	Cat# D2500-2
Index	Hampton Research	Cat# HR2-144
Crystal Screen	Hampton Research	Cat# HR2-110
Crystal Screen 2	Hampton Research	Cat# HR2-112
Natrix	Hampton Research	Cat# HR2-116
PEG/Ion Screen	Hampton Research	Cat# HR2-126
Structure Screen 1	Molecular Dimensions	Cat# MD1-01
Structure Screen 2	Molecular Dimensions	Cat# MD1-02
The Stura Footprint Screen	Molecular Dimensions	Cat# MD1-20
MacroSol	Molecular Dimensions	Cat# MD1-22
ProPlex Screen	Molecular Dimensions	Cat# MD1-38
The PACT Suite	QIAGEN	Cat# 135701-135796

**Deposited data**		

ZBTB7A(382-506)-C-194A DNA	This paper	PDB: 7EYI (https://www.rcsb.org/structure/7EYI)

**Oligonucleotides**		

See Table S1 for Oligonucleotides		

**Recombinant DNA**		

pcDNA3.1	Thermo Fisher Scientific	Cat#V79020
pcDNA3-FLAG-ZBTB7A residues 370-500	This paper	N/A
pcDNA3-FLAG-ZBTB7A residues 370-437	This paper	N/A
pcDNA3-FLAG-ZBTB7A residues 405-465	This paper	N/A
pcDNA3-FLAG-ZBTB7A residues 433-500	This paper	N/A
pcDNA3-FLAG-ZBTB7A residues 433-584	This paper	N/A
pcDNA3-FLAG-ZBTB7A residues 370-465	This paper	N/A
pcDNA3-FLAG-ZBTB7A residues 405-500	This paper	N/A
pcDNA3-FLAG-ZBTB7A residues 405-584	This paper	N/A

**Software and algorithms**		

HKL2000	(Otwinowski et al., 2003)	https://hkl-xray.com
SHELX	(Fu et al., 2007)	http://shelx.uni-goettingen.de/
CCP4	(Collaborative Computational Project, 1994)	https://www.ccp4.ac.uk
COOT	(Emsley and Cowtan, 2004)	https://openwetware.org
PHENIX	(Terwilliger et al., 2009)	http://www.phenix-online.org/
PyMOL	DeLano Scientific LLC	https://pymol.org/
MicroCal PEAQ-ITC	Malvern Panalytical	https://www.malvempanalytic.com

**Other**		

Hoefer™ SE 400 Series Sturdier™ Air-Cooled Vertical Electrophoresis Systems	Thermo Fisher Scientific	Cat# 10586935
Model 583 Gel dryer	Bio-Rad	Cat# 1651745
Typhoon™ FLA 9500 biomolecular imager	GE Healthcare	FLA 9500
MicroCal PEAQ-ITC	Malvern Panalytical	Serial: MAL1219954
High-Pressure Cell Disrupter	Shanghai LITU Co., Ltd	Cat# FB-110X
AKTA Purifier 10	GE Healthcare	Discontinued
Glutathione Sepharose 4B	GE Healthcare	Cat# 17-0756-01
HiLoad^TM^ 16/600 Superdex^TM^ 75 pg	GE Healthcare	Cat# 17-1068-01
Amicon® Ultra-15 Centrifugal Filter Unit (3 KDa MWCO)	Millipore	Cat# UFC900324
0.22 μm Syringe filter	Millipore	Cat# SLGPR33RB
0.22 μm Nitrocellulose Membrane	Millipore	Cat# GSWP04700
Nalgene Bottle top Filters	Nalgene company	Cat# 320-5045

## Materials and equipment

### Preparation of stock solutions (for EMSA)

**Timing: 5 h**

**Table:** Buffer A: Store at 4°C for up to 6 months

Reagent	Final concentration	Amount
1 M HEPES (pH 7.8)	10 mM	5 mL
1 M MgCl_2_	1.5 mM	750 μL
2 M KCl	10 mM	2.5 mL
Milli-Q water	N/A	491.8 mL
**Total**	**N/A**	**500 mL**

**Table:** Buffer C: Store at 4°C for up to 6 months

Reagent	Final concentration	Amount
1 M HEPES (pH 7.8)	20 mM	2 mL
100% glycerol	25%	25 mL
5 M NaCl	420 mM	8.4 mL
1 M MgCl_2_	1.5 mM	150 μL
0.5 M EDTA	0.2 mM	40 μL
Milli-Q water	N/A	64.4 mL
**Total**	**N/A**	**100 mL**

**Table:** 10× TNE buffer: 10× TNE can be freshly prepared, or a 50 mL stock can be prepared and stored for up to 3 years at room temperature (between 20°C–25°C)

Reagent	Final concentration	Amount
1 M Tris (pH 8.0)	100 mM	5 μL
5 M NaCl	500 mM	5 μL
0.5 M EDTA	10 mM	1 μL
Milli-Q water	N/A	39 μL
**Total**	**N/A**	**50 μL**

**Table:** 10× Gel shift buffer: Store for up to 3 years at room temperature (between 20°C–25°C)

Reagent	Final concentration	Amount
1 M HEPES (pH 7.8)	100 mM	5 mL
2 M KCl	500 mM	12.5 mL
1 M MgCl_2_	50 mM	2.5 mL
0.5 M EDTA	10 mM	1 mL
100% glycerol	50%	25 mL
Milli-Q water	N/A	4 mL
**Total**	**N/A**	**50 mL**

**Table:** 10× TBE buffer: Store for up to 6 months at room temperature (between 20°C–25°C)

Reagent	Final concentration	Amount
Tris	900 mM	108 g
Boric acid	900 mM	55 g
EDTA	50 mM	19.3 g
Milli-Q water	N/A	1 L
**Total**	**N/A**	**1 L**

• 25% (w/v) ammonium persulfate (APS): Dissolve 250 mg APS into 1 mL Milli-Q water. Store at 4°C for up to 1 month.
• 1 mg/mL poly dIdC: Add 1 mL Milli-Q water per 13 U poly dIdC stock powder (assuming product is approximately 13 U/mg; the exact amount is lot-specific). Invert to mix then transfer contents to a 15 mL conical tube. Sonicate for 3 min (30 s ‘on’/30 s ‘off’ on ‘high’; non-refrigerated). Aliquot stock solution into 1.5 mL microcentrifuge tubes and heat to 100°C for 1 min then let cool slowly to room temperature. Store for up to 3 years at −20°C.

### Preparation of stock solutions (for crystallographic analysis)

**Table:** Luria-Bertani (LB) medium: Autoclave the medium at 121°C for 20 min; store at room temperature

Reagent	Final concentration	Amount
NaCl	10 g/L	10 g
Yeast extract	5 g/L	5 g
Tryptone	10 g/L	10 g
Milli-Q water	N/A	1 L
**Total**	**N/A**	**1 L**

• Prepare the following antibiotics, IPTG, TCEP and protease inhibitor cocktail stock solutions.
  ○ 1 M IPTG: dissolve 7.14 g IPTG in ∼25 mL Milli-Q water, in a final volume of 30 mL. Filter the solution with a 0.22 μm syringe filter. Aliquot 1 mL/tube and store at −20°C.
  ○ 100 mg/mL Ampicillin: dissolve 4 g Ampicillin in ∼30 mL Milli-Q water, in a final volume of 40 mL. Filter the solution with a 0.22 μm syringe filter. Aliquot 1 mL/tube and store at −20°C.
  ○ 0.1 M TCEP: Dissolve 0.0287 g TCEP in ∼0.5 mL Milli-Q water, adjust pH to 7.5 with 2 M NaOH, to a final volume of 1 mL. Filter the solution with a 0.22 μm syringe filter. Aliquot 0.2 mL/tube and store at −20°C.
  ○ 1 tablet/1 mL protease inhibitor cocktail: Dissolve one tablet cOmplete, Mini, EDTA-free EASYpack in 0.8 mL Milli-Q water, to a final volume of 1 mL. Aliquot 0.25 mL/tube and store at −20°C.
• Prepare stock buffers for LR medium.
  ○ 1 M MgSO_4_: Dissolve 12.04 g MgSO_4_ in ∼90 mL Milli-Q water, to a final volume of 100 mL. Filter the solution with a 0.22 μm bottle top filter and autoclave at 121°C for 20 min. Store at 4°C.
  ○ 1 M ZnSO_4_: Dissolve 16.15 g ZnSO_4_ in ∼90 mL Milli-Q water, to a final volume of 100 mL. Filter the solution with a 0.22 μm bottle top filter and autoclave at 121°C for 20 min. Store at 4°C.
  ○ 1 M CaCl_2_: Dissolve 11.10 g CaCl_2_ in ∼90 mL Milli-Q water, to a final volume of 100 mL. Filter the solution with a 0.22 μm bottle top filter and autoclave at 121°C for 20 min. Store at 4°C.
  ○ 50% (w/v) Glucose: Dissolve 50 g glucose in ∼80 mL Milli-Q water, to a final volume of 100 mL. Filter the solution with a 0.22 μm bottle top filter and autoclave at 115°C for 30 min. Store at 4°C.
• LeMaster and Richards minimal medium (LR medium).
  ○ Dissolve 24 g KH_2_PO_4_, 5 g NaOH and 0.5 g NH_4_Cl in 980 mL Milli-Q water and adjust pH to 7.0 with H_2_SO_4_. Autoclave at 121°C for 20 min. The following reagents are prepared independently: 1 M CaCl_2_, 1 M MgSO_4_ and 1 M ZnSO_4_ are autoclaved at 121°C for 20 min. 50 g glucose is dissolved in 100 mL Milli-Q water and then autoclaved at 115°C for 30 min. Add 100 μL CaCl_2_, ZnSO_4_, 2.2 mL MgSO_4_ and 5 mL glucose into LR medium before use.
• GST binding buffer for GST-ZBTB7A-ZF1-4.
  ○ GST binding buffer for GST-ZBTB7A-ZF1-4 contains 20 mM Tris-HCl, pH 7.5, 1 M NaCl. Prepare 1 L and store at 4°C. Add DNase I and protease inhibitor cocktail stock before use.
• GST elution buffer: Freshly make before use.

**Table:** 

Reagent	Final concentration	Amount
Tris-HCl, pH 7.5	20 mM	N/A
NaCl	1 M	N/A
Reduced glutathione	30 mM	N/A
Milli-Q water	N/A	100 mL
**Total**	**N/A**	**100 mL**

• TEV enzyme cleavage buffer.
  ○ TEV enzyme cleavage buffer contains 20 mM Tris-HCl, pH 7.5, 400 mM NaCl. Freshly prepare 1 L and store at 4°C.
• Gel filtration buffer.
  ○ Gel filtration buffer contains 20 mM Tris-HCl, pH 7.5, 1 M NaCl. Freshly prepare 1 L and remove any bubbles by ultrasonication.
• DNA binding buffer.
  ○ DNA binding buffer contains 20 mM Tris-HCl, pH 7.5, 150 mM NaCl. Freshly prepare 1 L and store at 4°C.
• ITC titration buffer.
  ○ ITC titration buffer contains 20 mM Tris-HCl, pH 7.5, 150 mM or 300 mM NaCl. Freshly prepare 500 mL and store at 20°C, then de-gas before use.

**CRITICAL:** All stock buffers need to be filtered using 0.22 μm filter membranes and can be stored at 4°C for up to 1 year.

## Step-by-step method details

### Electrophoretic mobility shift assays (EMSAs)

***Note:*** The EMSA protocol is based on the one described by Crossley et al. (1996).

#### COS-7 cell transfections

**Timing: 4 days**

In this section mammalian cells are transfected with constructs encoding the protein of interest to drive transient overexpression so that the proteins can be harvested in the next section.

The cDNA encoding the ZBTB7A ZFs were previously cloned into the pcDNA3 mammalian expression plasmid and contained an N-terminal FLAG tag (Martyn et al., 2018; Yang et al., 2021). pcDNA3-FLAG-ZBTB7A ZF plasmid constructs were transiently overexpressed in COS-7 cells. After 48 h, cells were harvested and nuclear extracts (NXT) prepared for subsequent EMSA experiments.

The nuclear extraction protocol is optimized for approximately 10^6^ COS-7 cells. A pcDNA3 ‘empty’ construct that did not contain the ZBTB7A ZF region or FLAG tag was also transfected to act as a negative control.

1. Grow COS-7 cells in 10 cm tissue culture plates at 37°C in a 5% CO_2_ water jacketed incubator prior to transfection.
  a. Cells are cultured using Dulbecco’s Modified Eagle Medium (DMEM) supplemented with 10% (v/v) fetal calf serum (FCS) and 1% penicillin-streptomycin-glutamate (PSG).
  b. During passaging, incubate COS-7 adherent cells with 0.05% trypsin-EDTA for 5 min at 37°C to dislodge the cells.
2. Seed COS-7 cells in 10 cm tissue culture plates approximately 24 h prior to transfection to ensure plates are at approximately 50%–60% confluency on the day of transfection.
  a. This allows enough live cells to take up the ZBTB7A ZF plasmids, as well as the desired conditions for the live/transfected cells to divide and ensure growth is not impeded by dead cells.
3. On the day of transfection (day 1), warm DMEM (supplemented with 10% FCS and 1% PSG) and Opti-MEM (no FCS, no PSG) to 37°C for about 15 min before transfecting.
4. In a PC2 biosafety cabinet, add 5 μL of 1 mg/mL pcDNA3 ‘empty’ plasmid construct (5 μg total) into a new labeled 1.5 mL microcentrifuge tube. Repeat this step for all ZBTB7A ZF constructs to be transfected.
5. To a new set of 1.5 mL microcentrifuge tubes, add 85 μL Opti-MEM and 15 μL FuGENE6 Transfection Reagent per transfection.
6. Incubate the FuGENE6/Opti-MEM at room temperature (RT) for 5 min.
7. Pipette the FuGENE 6/Opti-MEM in one tube into the labeled tube containing 5 μg of plasmid drop-wise (make sure the FuGENE 6/Opti-MEM solution falls directly on top of the plasmid solution and does not adhere to the sides of the tube).
8. Incubate the FuGENE 6/Opti-MEM/plasmid mixtures at RT for 15 min.
9. During the 15 min incubation, replate the media on the COS-7 transfection plates with 10 mL of new, warmed DMEM media (supplemented with 10% FCS and 1% PSG).
  a. Label plates with the name of plasmid construct to be used. Leave COS-7 cells in the incubator until ready to add the FuGENE 6/Opti-MEM/plasmid mixture.
  b. Just before the end of the 15 min incubation, remove the 50%–60% confluent COS-7 plates from the incubator and place them in a PC2 biosafety cabinet.
10. After the 15 min incubation, add the total volume in the FuGENE 6/Opti-MEM/plasmid tube to the COS-7 plates at RT. Do this dropwise in a spiral pattern to ensure homogeneous distribution of transfection agent across the plate of cells.
11. Put the lid back on the plate and gently push the plate forward-and-back and side-to-side to evenly distribute the transfection mixture in the media.
12. Repeat steps 10 and 11 for any other ZBTB7A ZF plasmids.
13. Place the COS-7 cells back in the incubator to grow at 37°C in 5% CO_2_ for approximately 48 h.

**CRITICAL:** Do not grow the transfected COS-7 cells for more than 72 h as the COS-7 cells will tend to overgrow and die.

#### Harvesting COS-7 cell transfections and nuclear extractions

**Timing: 1.5 h**

In this section the transiently overexpressed proteins are extracted from the COS-7 cells. The nuclear extraction protocol is based on the one described by Andrews and Faller (1991).

14. Discard the DMEM media from the COS-7 transfection plates. The COS-7 cells do not need to be kept sterile from this point onwards.
15. Add 1 mL ice-cold PBS to each plate.
16. Scrape all the COS-7 cells off the surface of each plate using a cell scraper and transfer the cells and PBS to a new, labeled 1.5 mL microcentrifuge tube. Keep harvested transfected COS-7 cells on ice.
17. Centrifuge at 300 × *g* for 5 min and remove the supernatant. Proceed to performing nuclear extractions.

**Pause point:** After removing the supernatant from the harvested cells, freeze cell pellets at −80°C until needed. Cell pellets can be stored at −80°C for up to 3 months.

**CRITICAL:** Keep all cell pellets, buffer A and buffer C on ice while performing the nuclear extractions.

18. For each mL of buffer A and buffer C to be used, add 5 μL 0.2 M PMSF (dissolved in isopropanol), 5 μL 1 M DTT, 10 μL 1 mg/mL leupeptin and 10 μL 1 mg/mL aprotinin.
19. Resuspend the cell pellet in 300–400 μL buffer A and incubate on ice for 10 min. The suggested buffer A volume to use is around 10× the cell pellet size.
20. Vortex for 10 s, then spin for 10 s in a tabletop mini centrifuge.
21. Remove all the supernatant (cytoplasmic extract).
22. Resuspend the cell pellet in 50–100 μL buffer C and incubate for 20 min on ice. The suggested buffer C volume to use is around 1–2× the cell pellet size for ZBTB7A ZF transfection pellets. If your nuclear pellet is large then add more buffer C to ensure efficient high salt nuclear protein extraction.
23. Pre-chill a centrifuge to 4°C and pre-chill new labeled 1.5 mL microcentrifuge tubes on ice during the 20 min incubation.
24. Centrifuge samples at 18,407 × *g* for 3 min at 4°C.
25. Transfer the nuclear extract (NXT) supernatant into the pre-chilled 1.5 mL microcentrifuge tube. Repeat for all samples. Keep all NXT on ice.

***Note:*** Determine protein concentration at this point if necessary, e.g., for Western blot, by measuring the absorbance at 280 nm on a spectrophotometer at 280 nm.

26. Proceed to EMSA sample preparation if DNA probes for EMSAs have already been radio-labeled and purified, and acrylamide gel(s) have been cast.

**Pause point:** NXTs can be stored at −80°C for up to 1 month. Thaw frozen NXT on ice before use. Avoid repeated freeze-thaw cycles as this can lead to the NXT proteins degrading.

#### Preparation of radiolabeled DNA probes

**Timing: 6 h**

Radiolabeled DNA probes are short complementary, double-stranded DNA oligonucleotides that are labeled using ATP [γ-^32^P]. The 5′ end of the sense (forward) oligonucleotide is labeled with ATP [γ-^32^P] using T4 PNK enzyme by incubating at 37°C for 30 min then boiling for 1 min at 100°C. The unlabeled antisense (reverse) strand is then added to the labeled forward oligonucleotide and annealed via slow cooling from 100°C to RT.

When annealing radiolabeled complementary oligonucleotides, only the forward strand is labeled, and not both the forward and reverse strands at the same time. T4 PNK kinase is more efficient at phosphorylating single-stranded DNA. Furthermore, background bands may be present in EMSAs due to the presence of radiolabeled single-stranded DNA binding to endogenous proteins. Addition of excess unlabeled reverse strand drives annealing efficiency to produce double-stranded DNA and ensure there will be very little single-stranded DNA to bind to endogenous proteins.

**Important**: The efficiency of radiolabeling probes is dependent on the residue occupying the 5′ end of the forward oligonucleotide. Avoid designing forward probes that begin with a 5′ cytosine residue. Forward oligonucleotides that start with a cytosine are labeled 4-fold less efficiently than oligonucleotides starting with adenine or thymine, and 6-fold less efficiently than oligonucleotides beginning with a guanine.

27. Dilute the wild-type (WT) -200γ and mutant -200γ forward and reverse DNA oligonucleotides (oligos) to a final concentration of 15 μM by adding 15 μL 100 μM oligo stock and 85 μL Milli-Q water in a 1.5 mL microcentrifuge tube to a total volume of 100 μL. The diluted 15 μM stocks can be stored at −20°C.
  a. Set up and label one new 1.5 mL microcentrifuge tube for each forward and reverse oligo.
28. Add 1 μL of 15 μM forward oligo to the new 1.5 mL microcentrifuge tube (the ‘forward’ tube), ensuring the liquid is at the very bottom of the tube.
29. To the other tube (the ‘reverse’ tube), add 32.3 μL Milli-Q water, 4 μL 10× TNE buffer and 3.7 μL of 15 μM reverse oligo.
30. Make an ‘n+1’ master mix to radiolabel the forward oligo. ‘n’ is the number of probes to be labeled.
  a. For each forward oligo to be radiolabeled add 6 μL Milli-Q water, 1 μL PNK buffer and 1 μL T4 PNK enzyme (10,000 units/mL) into a 1.5 mL microcentrifuge tube.
  b. The tube containing the master mix can be placed into a portable cold cooling block or a container with ice to keep the T4 PNK enzyme cold.

**CRITICAL:** Perform the next steps in a certified radiation facility. Additional PPE such as laboratory-grade safety glasses, lab coats and disposable gloves must be worn. Equipment and reagents such as Geiger Counters, Decon 90, Perspex containers that can hold 1.5 mL microcentrifuge tubes and a Perspex container double-lined with bin liners for radioactive waste are required.

***Note:*** The use of long forceps is encouraged to transfer tubes containing ^32^P as this decreases radiation exposure by increasing the distance between your body and the radiation. Wearing two layers of gloves is also encouraged when handling ^32^P in case of radioactive contamination on the outer gloves and/or breakage of the outer layer of gloves.

31. Set a heat block to 37°C.
  a. Place the forward oligo master mix into a Perspex box that can hold 1.5 mL microcentrifuge tubes and open the tube lid carefully.
32. Add ‘n+1’ volume of 1 μL ATP [γ-^32^P] (250 μCi) to the forward oligo master mix.
  a. Dispose of the pipette tip and all other radioactively-contaminated materials into the double-lined Perspex waste container.
33. Close the tube lid firmly, spin the tube quickly in the tabletop mini centrifuge to ensure all liquid is at the bottom of the tube and transfer it back to the Perspex tube container.
  a. Line up all forward probe tubes containing 1 μL of 15 uM forward oligo into the Perspex tube container and open the lids.
34. Aliquot 9 μL of the forward probe master mix into each of the forward oligo tubes. Use a new pipette tip for each tube. Dispose the remaining master mix.
  a. Close all lids firmly, spin quickly in the tabletop mini centrifuge and carefully transfer tubes back to the Perspex container.
35. Incubate forward oligos for 30 min at 37°C in the heat block. Place 1.5 mL microcentrifuge tube cap locks on the lids to prevent lids from opening in the next step.

***Note:*** Use a Perspex shield or place an upside-down open Perspex container on top of the heat block to shield from any radioactivity.

**Figure 1:**
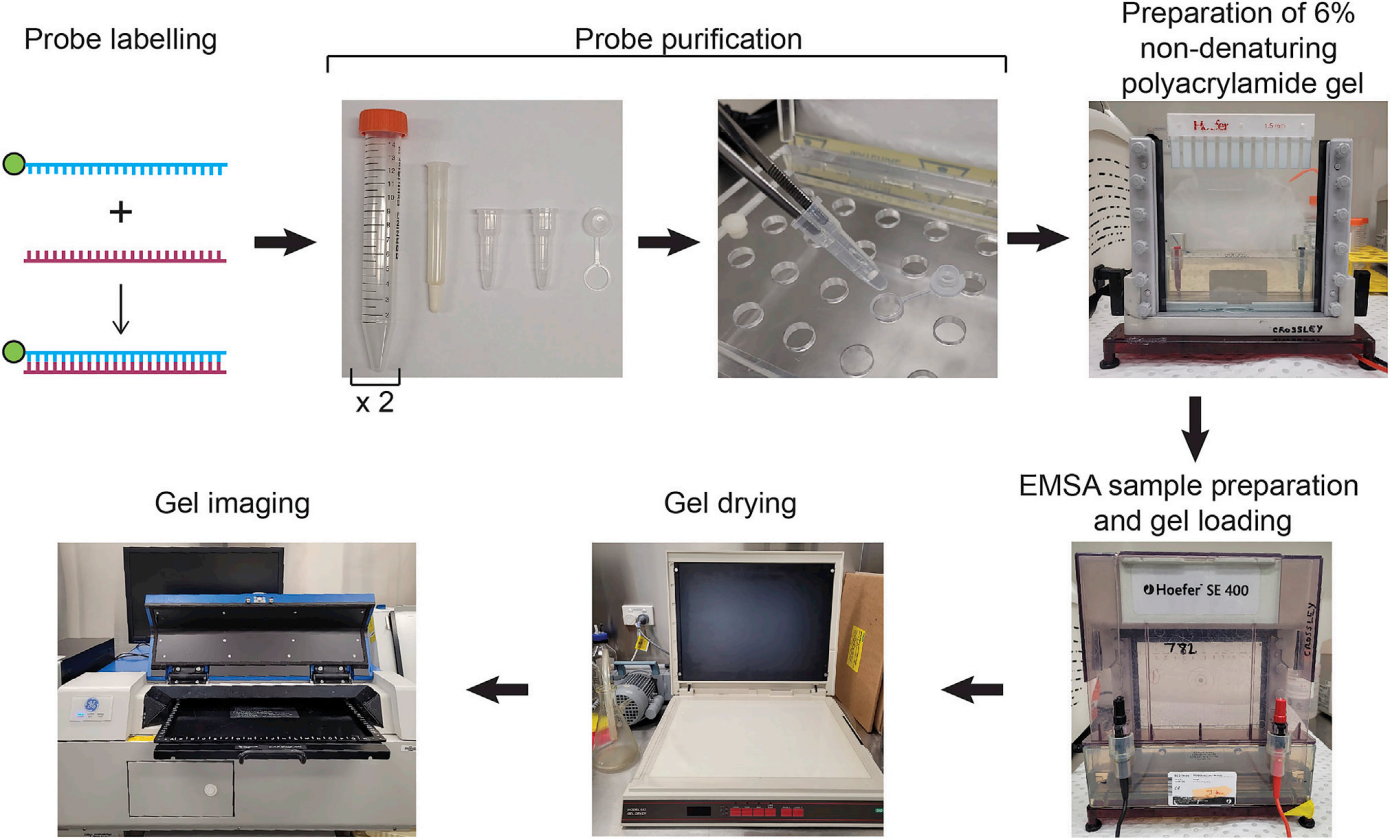
Procedure for performing electrophoretic mobility shift assays using a radiolabeled DNA probe to analyze ZBTB7A ZF-DNA binding The experimental steps include DNA probe labeling with ATP [γ-^32^P], probe purification, the preparation of a 6% non-denaturing polyacrylamide gel, EMSA sample preparation and loading, gel drying and gel imaging.

36. After 30 min has elapsed, set the heat block to 100°C whilst the tubes remain in the heat block.
37. Boil the forward probes for 1 min at 100°C. It will take around 10–15 min to heat up to 100°C.
38. Carefully remove the cap locks, immediately pressing firmly on the tube lids to ensure they remain closed. Transfer the tubes to the Perspex tube container. Leave the heat block on at 100°C.
39. Spin all forward oligo tubes in the tabletop mini centrifuge for at least 10 s to ensure no ^32^P is on the inner tube lid.
40. Keeping the tube in the Perspex tube container, carefully open the forward oligo tube. Check for radiation contamination on fingers and thumb using a Geiger counter. Remove and change gloves if contamination occurs.
41. Add 40 μL of the reverse oligo mixture into the corresponding forward oligo tube and close the lid. Spin tubes in the tabletop mini centrifuge and transfer back to the Perspex box.
42. Place tubes back in the heat block, put tube cap locks on the lids and set the temperature of the heat block to room temperature (20°C–25°C). Leave tubes to cool down to room temperature (around 4–6 h) to allow radiolabeled forward oligo and reverse oligo to anneal (Figure 1).

**Pause point:** Tubes can be left overnight (∼ 12–16 h) to cool and probes can be purified the following day.

#### Probe purification

**Timing: 2 h**

In this section the radiolabelled probes are purified prior to mixing them with the proteins of interest to assess binding.

43. Gather Quick Spin Columns for radiolabeled DNA purification (Sephadex G-25 columns, *Sigma*-*Aldrich company*) (stored at 4°C) to purify the radiolabeled probes. For each probe you will need two collection tubes, one lid, one column and two 15 mL conical tubes (conical tubes not included in the Sephadex G-25 column kit) (Figure 1).
44. Shake or vortex the columns to break up the material inside to ensure even distribution.
45. Open the top of the column then remove the bottom. Place bottom of the column into a collection tube and place the column and collection tube into the 15 mL conical tube. Repeat for all columns.
  a. Label the remaining collection tubes with the probe names.
46. Centrifuge the 15 mL conical tubes at 1,100 × *g* for 1.5 min to collect the liquid from the column in the collection tube.
47. Open the lid of the 15 mL conical tube and use tweezers to remove the column then the collection tube. Discard the liquid in the collection tube. Reassemble the collection tube, column and 15 mL conical tube.
48. Centrifuge the 15 mL conical tube again for 1,100 × *g* for 1.5 min.
49. Insert the dried column into the new, labeled collection tube and place the dry column/collection tube into a new 15 mL conical tube.

***Note:*** The powder in the column should appear dry and chalky after the second spin. If it still appears moist, centrifuge again to remove all residual liquid.

50. Spin cooled radiolabeled probes in tabletop mini centrifuge to collect liquid at the bottom of the tube.
51. Open the lid of the 15 mL conical tube and transfer the entire radiolabeled probe (50 μL) onto the top of the column. Ensure you do not touch any other part of the column set up with the pipette tip.
52. Place the conical tube lid back on and centrifuge at 1,100 × *g* for 4 min.
  a. During the spin, place the column lids on the Perspex tube container by aligning the ring of the collection tube lid with the opening of the Perspex box tube hole (Figure 1).
53. Use long forceps to carefully remove and discard the dried column.
54. Use long forceps to carefully transfer the collection tube containing the purified probe into the ring (Figure 1). Press the collection tube firmly down into the ring, close the lid and place purified probes in at 4°C. Check the forceps for any radioactive contamination between transferring each probe.

**Pause point:** Proceed immediately to non-denaturing polyacrylamide gel set up or continue another day. ^32^P has a half-life of 14.3 days.

#### Preparation of 6% polyacrylamide gel

**Timing: 1 h**

In this section a native polyacrylamide gel is poured so that it can be used to load and separate probe-protein complexes in later sections.

55. Clean two glass plates (18 × 16 cm) with absolute ethanol.
56. Place two spacers (1.5 mm thickness, 2 cm width, 16 cm height) at either end of one of the glass plates. Place the second glass plate over on top, ensuring plates are flush with the spacers.
57. Screw on clamp assemblies (16 cm) at either end of the two glass plates where the spacers are inserted.
58. Place the set up into the casting stand opposite the lower buffer chamber, and secure with clips. When inserting and turning the clips, there should be increasing resistance as the glass plates and clamp assemblies are locked into the casting stand.
59. Prepare the acrylamide gel (6%) by adding the following reagents, in order, to a 50 mL conical tube:

**Table:** 

Reagent	Amount
Milli-Q water	39 mL
10× TBE	2.5 mL
40% acrylamide/bis-acrylamide (19:1)	7.5 mL
25% APS	200 μL
TEMED	50 μL

***Note:*** Add 25% APS and TEMED immediately before pouring the gel. Close the lid of the 50 mL conical tube and invert to mix (do not resuspend with a graduated pipette in case of spillage). If casting more than one gel, prepare each gel individually so the acrylamide does not polymerize in the tube once the APS and TEMED are added. Proceed with caution when handling acrylamide as it may cause nerve damage in humans. Always wear PPE such as a lab coat, safety glasses and two layers of gloves when handling acrylamide. Wash skin thoroughly if contact occurs with skin.

60. Using a graduated pipette, pour the acrylamide solution immediately into the gel casting cassette (between the two glass plates, and add a 12-well comb between the glass plates (Figure 1) (see troubleshooting 1 and 2). Ensure there are no bubbles on the base of the comb or in the gel.
  a. Mark the comb position and number each lane with a marker.
  b. When the acrylamide has polymerized, transfer the gel apparatus to a 4°C fridge to chill for 30–40 min.
61. Prepare 0.5× TBE buffer by adding 25 mL 10× TBE and 475 mL Milli-Q water. 500 mL 0.5× TBE is required per EMSA. Place buffer at 4°C.

***Note:*** Proceed to preparing EMSA master mix while gels are polymerizing.

#### EMSA sample preparation and gel loading

**Timing: 20–30 min**

In this section the proteins of interest (nuclear extracts) and the radiolabelled probes are combined and then loaded onto the native polyacrylamide gel for separation.

62. Place a cooling block on ice and prepare one labeled 1.5 mL microcentrifuge tubes for each sample to run on the EMSA.
63. Prepare EMSA sample mix as follows:

**Table:** 

Reagent	Final concentration	Amount
1 mg/mL poly dIdC	0.05 mg/mL	1.5 μL
1 mg/mL BSA	0.1 mg/mL	3 μL
10 mM DTT	1 mM	3 μL
10× gel shift buffer	1×	3 μL
Nuclear extracts (NXT)	N/A	5 μL
Milli-Q water	N/A	13.5 μL

**Important**: Keep all NXT and gel shift sample mixes on ice.

***Note:*** NXT and Milli-Q water amount can be varied accordingly to the NXT concentration. If the same NXT is used for multiple samples, prepare a ‘n+1’ master mix in a 1.5 mL tube (where ‘n’ is the number of samples) and aliquot 29 μL into each tube.

***Optional:*** An additional sample that has an antibody that binds to the protein of interest, such as an anti-FLAG antibody, can be used to confirm the identity of the protein of interest bound to the probe. This is referred to as a ‘supershift’ as addition of antibody to the sample retards the protein:probe complex in the polyacrylamide gel. Generally, 1 μL of crude antisera or commercially available antibody is added to the supershift sample in addition to the components above (although different amounts can be tested). The antibody may be added during the EMSA sample preparation (before addition of radiolabeled probe) or after the addition of the radiolabeled probe.

***Note:*** Addition of the antibody before the radiolabeled probe may interfere with binding of the protein of interest to the probe, addition after is more likely to lead to a ‘supershift’.

64. Remove radiolabeled probes from the 4°C fridge and spin them down in the tabletop mini centrifuge for at least 10 s.
65. Add 1 μL of desired probe to each of the EMSA samples. Spin EMSA samples in the tabletop mini centrifuge.
66. Place the samples back on the cooling block and incubate at 4°C for 10 min. This allows binding of probe to the protein of interest.
  a. During the 10 min incubation, remove the acrylamide gel from the fridge.
  b. Remove the clips holding the gel in place in the casting stand and transfer the gel set up to the lower buffer chamber.
  c. Fill bottom chamber with 250 mL 0.5× TBE, remove the comb and flush out the wells using a syringe fixed with a blunt needle. Repeat for all gels. Place gels (with 0.5× TBE in the lower buffer chamber) back at 4°C. When ready to load the samples, remove the gels (in the gel tanks) from the fridge.
67. Spin EMSA samples in the tabletop mini centrifuge and load samples (∼30 μL) into the wells of the acrylamide gel.

***Note:*** If there are surplus lanes in the gel, 10–20 μL 6× DNA loading dye can be added to observe migration during electrophoresis.

68. Place the upper buffer chamber on top of the gel and secure with clips. There should be increasing resistance when tightening the clips.
69. Pour 250 mL 0.5× TBE in the upper buffer chamber and place the safety lid with electrodes over the entire apparatus (Figure 1).
70. Carefully transfer complete gel apparatus to the 4°C fridge, plug the electrodes into a power pack and run gel for 1 h 45 min at 250 V at 4°C.
  a. Check that the gel is running after 1–2 min.
  b. Ensure you have at least 1 sheet of thick (1 mm) filter paper per gel. Two pieces of thinner filter paper can be used instead if thicker filter paper is not available.
  c. Turn on the gel dryer about 10 min before gel electrophoresis is due to finish. Set the temperature to 80°C and allow it to heat up. Ensure the vacuum pump is working.

***Note:*** The electrophoresis duration described here is based on using probes that are 23 bp in length. If using shorter probes, run electrophoresis for a shorter time to ensure the probe does not migrate off the gel. Similarly, if using longer probes, run electrophoresis for a longer duration to allow better separation of bands. This may require some trial-and-error when performing this for the first time. Alternatively, the percentage of polyacrylamide in gel may be changed, or agarose gels may be used instead of polyacrylamide gels.

#### Gel drying

**Timing: 45 min**

In this section the native polyacrylamide gel is dried so that it can be imaged and the protein-probe complexes visualized in the next section.

71. Once electrophoresis is complete, remove the gel tank from the fridge. Remove the safety lid, upper buffer chamber and lower buffer chamber. Discard the TBE liquid waste into the appropriate waste.
72. Use a plate separation tool to slide out one of the spacers and slowly open the glass plates, lifting one plate off from the gel. Ensure the gel remains flat on one glass plate and does not shift or fold back on itself.
73. Place a piece of filter paper over the gel, pat down and lift the paper up (or flip the filter paper and glass plate over) to peel the gel off. The gel should be attached to the filter paper.
  a. If using thinner paper, place one more piece of filter paper behind the first piece of filter paper.
  b. Mark the position of lane 1 on the filter paper using a pencil/pen.
74. Place cling film over the surface of the gel. Do not wrap the cling film underneath the filter paper or it will interfere with the drying process.
75. Place the gel on the gel dryer with the filter paper on the bottom and cover the gel(s) with the gel dryer plastic cover. Turn on the vacuum pump and ensure the plastic cover of the gel dryer forms a suction seal around the perimeter of the gel dryer surface. If not, turn off the gel dryer, readjust the dryer surface and gels and try again.
  a. Dry gel for 30 min for one gel or 45 min for two or more gels. The gels should appear very flat and shiny after drying. If not completely dry, continue drying for another 10–20 min.
  b. “Blank” one phosphor screen per gel by placing it under white light for 30 min. Ensure the phosphor screen fits into the gel cassette.
76. When gels are completely dry, place the gel (now stuck to the filter paper) and the cling film into a gel cassette.
77. Place the white side of the phosphor screen onto the gel and close the cassette. Avoid shifting the phosphor screen on the gel.
78. Leave gels overnight (12 h–24 h) on a stationary surface.

***Note:*** Phosphor screens can be imaged after 2 h, however the image quality will improve with longer exposure times.

#### Gel imaging

**Timing: 30 min**

In this section the gels are imaged so that the degree of binding of the protein to the radiolabelled probes can be determined.

79. Turn on phosphor imager.
80. Open cassette and transfer black side (magnetic side) of the phosphor screen onto the screen holder. Take note of the coordinates where you placed the phosphor screen on the screen holder.

***Note:*** If imaging multiple phosphor screens, phosphor screen can be imaged individually or all together on the same screen holder.

81. Place screen holder into the phosphor imager and close the phosphor imager lid.
82. Open the phosphor imaging software:
  a. Name your file (and pre-save it if necessary).
  b. Set the coordinates for where the phorphor imager should scan the screen holder.
  c. Set the filter to IP.
  d. Set pixel size to 100 μM and PMT voltage to 700 V.
83. Scan the phosphor screen and perform subsequent image manipulation on software such as ImageJ or ImageLab (see troubleshooting 3, 4, and 5).

### Crystallographic analysis

#### Preparation for the expression plasmid and strain for ZBTB7A

**Timing: 3–4 days**

In this section the process of generating the expression plasmid encoding the protein of interest by cloning is described.

84. Amplify the C2H2 zinc finger coding region of human ZBTB7A (NCBI Reference Sequence: NP_001304919.1; residues 382–506) with an additional tryptophan (W) at the end of the C-terminus by PCR from human bone marrow cDNA library (BD Clontech). Digest the PCR product with the restriction enzymes *Nde*I and *Xho*I. Clone the digested product into a modified pGEX-4T1 vector (*GE Healthcare*) that contains a TEV protease cleavage site (amino acid sequence: ENLYFQG) after the GST tag (pGEX-4T1-TEV). The sequences of primers used are listed in key resources table.
  ***Note:*** The concentration of proteins is usually determined spectrophotometrically at 280 nm. However, the extinction coefficient of ZF1-4 of ZBTB7A is 0.442 M^-1^ cm^-1^, which is difficult to determine the precise concentration of. Addition of a W at the C-terminus end increases the extinction coefficient of ZBTB7A ZF1-4 to 0.802 M^-1^ cm^-1^, which is high enough to determine the concentration for. Moreover, further crystallography experiments demonstrated that the extra W has no influence in crystallization and DNA binding.
  a. The reaction mixture for PCR amplification of the target gene fragment is indicated below.

**Table:** 

Reagent	Final concentration	Amount
Bone marrow cDNA	N/A	1 μL
Primer forward (10 μM)	0.2 μM	1 μL
Primer reverse (10 μM)	0.2 μM	1 μL
Sterile water	N/A	22 μL
PrimeSTAR Max DNA Polymerase (2×)	1×	25 μL
Total	N/A	50 μL

    ***Note:*** The primers are synthesized in powder form. Dissolve primers with sterile water to 10 μM then centrifuge at 15,871 × *g* for 1 min. PrimeSTAR Max DNA Polymerase (2×) is stored at −20°C and thawed/kept on ice when in use. Spin the reaction mixture in a tabletop mini centrifuge to get rid of any bubbles before running the PCR.
  b. The PCR thermocycling parameters are outlined below.

**Table:** 

Steps	Temperature	Time	Cycles
Initial denaturation	98°C	5 min	1
Denaturation	98°C	20 s	32
Annealing	55°C	20 s	
Extension	72°C	30 s	
Final extension	72°C	5 min	1
Hold	4°C	Indefinitely

    ***Note:*** The extension time for the cycling depends on the efficiency of the DNA polymerase (10–15 s/kb) and the length of DNA fragment. The DNA fragment of interest is ∼380 bp, so a 30 s extension time is sufficient.
85. Run PCR products on a 1% agarose gel and purify the target DNA fragment by using E.Z.N.A. Gel Extraction Kit (Omega) according to the manufacturer’s protocol (https://ensur.omegabio.com/ensur/contentAction.aspx?key=Production.1999.S2R4E1A3.20181107.32.4668301). Determine the concentration of DNA fragment by measuring the absorbance at 260 nm.
86. Digest the PCR product with the restriction enzymes *Nde*I and *Xho*I, and ligated into a pGEX-4T1-TEV vector. The pGEX-4T1-TEV vector is a pGEX-4T1 vector (*GE Healthcare*) that contains a TEV protease cleavage site (amino acid sequence: ENLYFQG) after the GST tag.
  a. Add 1.5 μL each of *Nde*I and *Xho*I restriction enzymes and 5 μL reaction buffer (10×) to the reaction mixture containing 1.5 μg of the PCR product.
  b. Gently mix by pipetting up and down and briefly spin down the reaction mixture to get rid of bubbles, then immediately incubate at 37°C for 1–2 h.
  c. The restriction enzyme digested PCR product is purified using the E.Z.N.A. Gel Extraction Kit (*Omega*).
  d. Ligate the digested PCR product into a *NdeI*/*XhoI*-digested pGEX-4T1-TEV vector using a DNA Ligation Kit (Version 2.1, *TaKaRa*). Prepare the reaction mixture for ligation (outlined below) and incubate at 16°C for 1–2 h.

**Table:** 

Reagent	Amount
Solution I (2×)	5 μL
Digested DNA product	4 μL
Digested vector	1 μL

***Note:*** The pGEX-4T1 vector should be digested with same NdeI and XhoI restriction enzymes before the ligation experiment.

87. Transform into BL21 (DE3) competent cells.
  a. Gently thaw the BL21 (DE3) cells, which are pre-packaged in 100 μL, tubes on ice.
  b. Add 10 μL ligation product into BL21 (DE3) cells and incubate on ice for 20–30 min.
  c. Heat-shock the tube at 42°C for 60 s, then incubate the tube on ice for 2 min.
  d. Add 0.5 mL Luria-Bertani (LB) medium to the tube and incubate the tube at 37°C in a shaking incubator for 30–40 min with shaking at 220 rpm. During the incubation, pre-warm an LB-Ampicillin agar plate at 37°C.
  e. Centrifuge the transformation mixture at 5,170 x g for 1 min. Discard 450 μL supernatant, resuspend the remaining transformation mixture in the tube gently with the pipette, and spread 100–150 μL of the resuspended cells on the LB-Ampicillin agar plate.
  f. Incubate the LB-Ampicillin transformation plates at 37°C overnight (∼ 12–16 h).

***Note:*** Do not touch the bottom of the tube with BL21 (DE3) competent cells and do not mix the ligation product with competent cells with the pipette.

88. Pick a single clone with a sterile pipette tip and place the tip into 5 mL LB medium supplemented with 100 μg/mL ampicillin. Incubate at 37°C with shaking at 220 rpm for 8 h.
89. Extract the plasmids from the cells using an AxyPrep Plasmid Miniprep Kit as per the manufacturer’s protocol (https://www.corning.com/catalog/cls/documents/protocols/RMI060002422.pdf).
90. Verify the construct by DNA sequencing using pGEX-3′ primer and store at −20°C for the subsequent experiments.

**CRITICAL:** Ensure that the DNA sequence of the target gene is correct. Additionally, ensure a stop codon (TAA) is included immediately after the target gene sequence.

#### Preparation of 13% sodium dodecyl sulfate polyacrylamide (SDS-PAGE) gel

**Timing: 2 h**

In this section a SDS-PAGE gel is prepared to allow the extent of protein overexpression to be determined.

91. Prepare the resolving gel (13%). Mix the reagents listed below in the following order:

**Table:** 

Reagent	Amount
Milli-Q water	0.6 mL
Glycerol, 80%	0.6 mL
Gel buffer	1.2 mL
40% acrylamide/bis-acrylamide (29:1)	1.2 mL
APS, 10%	20 μL
TEMED	2 μL
Total	∼3.6 mL

***Note:*** Gel buffer (pH 8.45) made in house (3 M Tris-HCl and 0.3% (w/v) SDS). TEMED and APS are added to the SDS-PAGE resolving gel solution right before pouring the gel.

92. Pour the mixture immediately into the gel casting cassette, leaving a ∼2 cm gap below the bottom of the comb for the stacking gel.
93. Layer the top of the gel with Milli-Q water. This will keep the polymerized gel from drying out. The gel should be completely polymerized within 20 min at RT.
94. Remove the Milli-Q water.
95. Prepare the stacking gel (5%) by mixing the reagents listed below in the following order:

**Table:** 

Reagent	Amount
Milli-Q water	1.0 mL
Gel buffer	0.4 mL
40% acrylamide/bis-acrylamide (29:1)	0.2 mL
APS, 10%	20 μL
TEMED	2 μL
Total	∼1.6 mL

96. Pour the stacking gel on top of the resolving gel.
97. Add the combs to make wells. The stacking gel will completely polymerize in 20 min. Remove the combs before proceeding to the next step.

#### Transformation and bacterial glycerol stock

**Timing: 2 days**

In this section the expression plasmid is transformed into BL21 (DE3) and a bacterial glycerol stock is made for future use.

98. Transform 50–100 ng of the expression plasmid that encodes the C-terminal ZF DNA binding domain (amino acids 382–506) of ZBTB7A into BL21 (DE3) competent cells. Plate the transformed sample onto LB-Ampicillin plate and incubate at 37°C for 12–16 h.
99. Pick a single clone using a sterile pipette tip and place into 5 mL LB medium supplemented with 100 μg/mL ampicillin. Incubate at 37°C with shaking at 220 rpm for 8 h. Add 800 μL cultured cells to 200 μL autoclaved 80% glycerol in a 1.5 mL tube and pipette up and down gently. Store at −40°C.

#### Expression and purification of ZBTB7A-ZF1-4

**Timing: 5 days**

In this section the ZBTB7A protein is prepared for crystallization, and further ITC experiments (Figure 3).

100. Inoculate 5 μL of glycerol stock using a sterile pipette tip into 5 mL LB medium supplemented with 100 μg/mL ampicillin. Incubate at 37°C with shaking at 220 rpm for 8 h. Transfer 1 mL of cultured bacteria into 100 mL LR medium, supplemented with 100 μg/mL ampicillin. Incubate at 37°C with shaking at 220 rpm overnight (∼ 12–16 h).
101. Transfer 50 mL LR medium into 1 L LR medium supplemented with 100 μg/mL ampicillin in 2 × 2.8-L Fernbach culture flasks. Incubate at 37°C with shaking at 220 rpm until OD_600_ reaches 1.0–1.2 (see troubleshooting 6). This usually takes 3–4 h.
102. Cool down the cultured cells in an ice-bath and add 200 μL 1 M IPTG to the flask to reach a final concentration of 0.2 mM. Reduce the shaker temperature to 16°C and keep incubating with shaking for 18–24 h.
103. Harvest cells by centrifuging at 3,590 × *g* for 10 min at 4°C and then discard supernatant. Gently wash the cells with Milli-Q water.
104. Resuspend the cell pellets in 40 mL GST binding buffer per L cell culture volume by pipetting up and down using a 5 mL transfer pipette. Transfer the resuspended cells to 50 mL conical tubes.
105. Add 1:100 (v/v) protease inhibitor cocktail and 20 μL DNase I (20 U) to the re-suspended cells.
106. Lyse the cells using a high-pressure cell disrupter for 2–3 min at 10 L/h rate.

***Note:*** The high-pressure cell disrupter should be washed with Milli-Q water and GST binding buffer at least twice, each. Additionally, pre-cool the high-pressure cell disrupter to 6°C before harvesting cells.

107. Spin down the cell lysate at 24,300 × *g* for 30 min at 4°C to remove all debris and insoluble fractions (see troubleshooting 7).

**CRITICAL:** Keep cell lysates on ice during protein purification.

108. At the same time, wash the 10 mL column filled with 3 mL Glutathione Sepharose 4B with at least 5 column volumes (CV) Milli-Q water and 5 CV GST binding buffer using a 5 mL transfer pipette.
109. Incubate the cell lysate supernatant with the Glutathione Sepharose 4B with rotation for 6 h at 4°C.
110. Load the mixture of the cell lysate supernatant and Glutathione Sepharose 4B into a GST column at a 1–2 mL/min flow rate.
111. Wash the GST column with at least 8 CV GST binding buffer.
112. Elute the target protein with 20 mL GST elution buffer, transfer to 50 mL conical tube and incubate for 2 h with rotation at 4°C.
113. Collect the flow through and repeat the step 15, collecting the flow through again.
114. Add 1 mg TEV enzyme (10 mg/mL and stored at −80°C at about 100 μL/tube) to the flow through and then dialyze in TEV enzyme cleavage buffer at 4°C overnight (∼ 12–16 h).
115. Evaluate the digestion efficiency of TEV enzyme by SDS-PAGE.
116. Concentrate the final flow through volume to ∼5 mL using an Amicon Ultra-15 Centrifugal Filter Unit (3 KDa MWCO) at a speed of 2,800 × *g* at 4°C.
117. Inject the concentrated sample onto HiLoad 16/600 Superdex 75 pg column pre-equilibrated with 1.5 CV gel filtration buffer and run at 1 mL/min. ZBTB7A-ZF1-4 elutes at ∼80 mL on this column.

**Figure 2:**
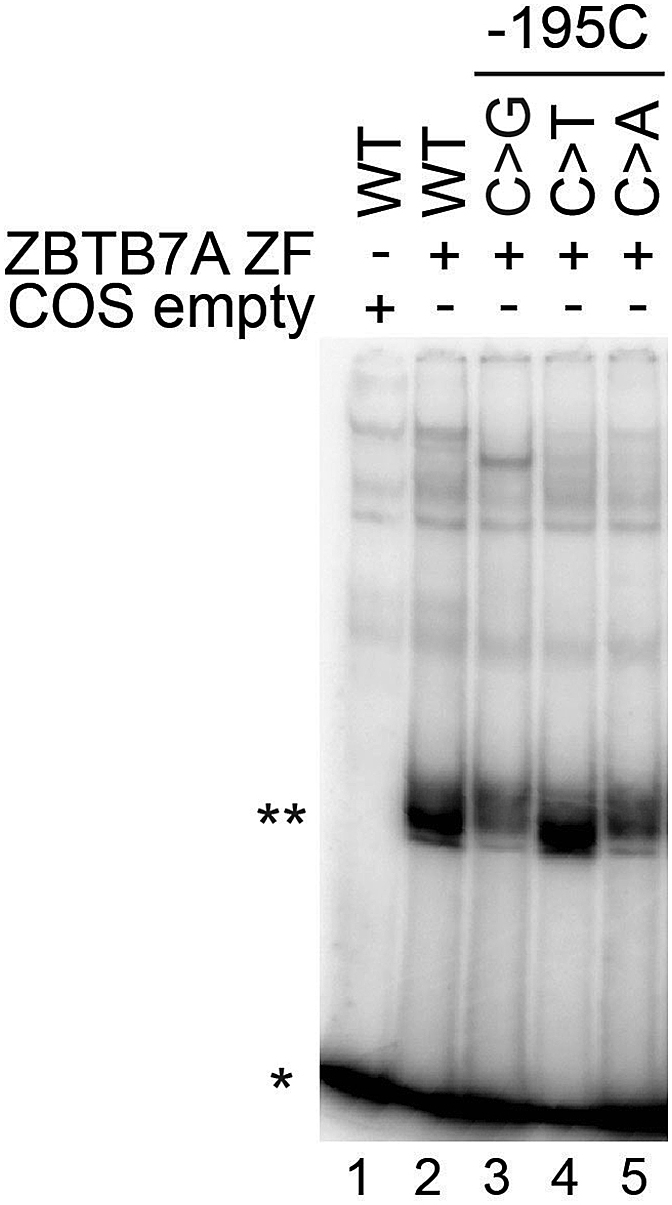
Example EMSA ZBTB7A ZF1-4 binds to the -200γ WT probe in lane 2 (∗∗). ZBTB7A ZF1-4 binding is reduced in lanes 3 and 5 with the use of mutant probes. A pcDNA3 ‘empty’ control was used in lane 1 to observe background binding of endogenous COS-7 proteins. Free probe that is not bound to any protein (∗) migrates to the bottom of the gel. If working with different ZBTB7A ZF truncations, DNA-probe bands may appear at different migration positions in the gel due to the smaller size of the truncated protein. Published in Yang et al. (2021).

**Figure 3:**
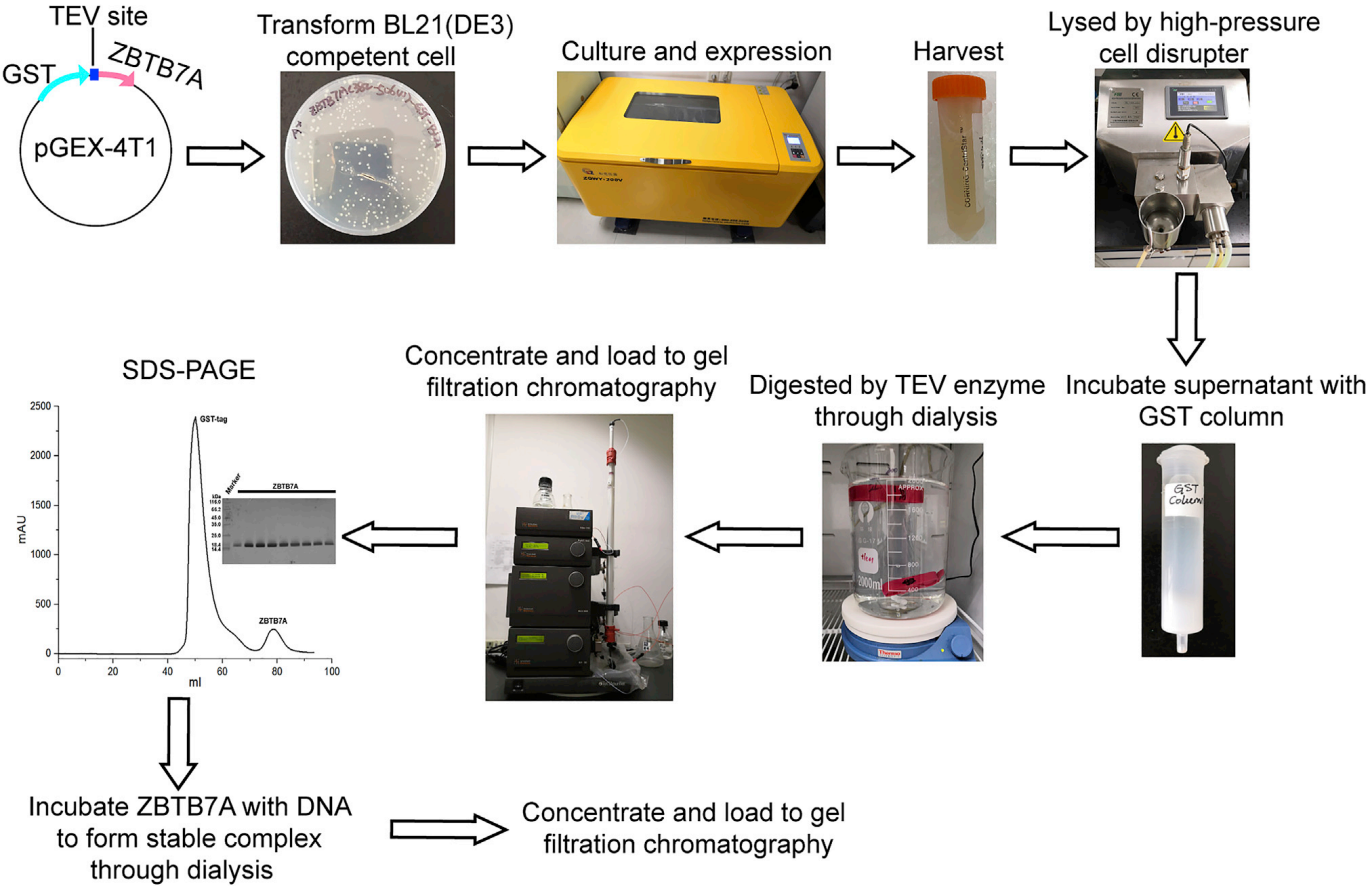
Procedures for the expression and purification of ZBTB7A and ZBTB7A-DNA complex The purification steps include lysis of cells by high-pressure cell disrupter, incubation of the supernatant with the GST column, cleavage by TEV enzyme through dialysis, and size exclusion chromatography for ZBTB7A and ZBTB7A-DNA complex, respectively. Use SDS-PAGE to evaluate the purity of protein or protein-DNA complex.

#### DNA annealing

**Timing: 1 day**

In this section the single-stranded oligonucleotides are annealed to each other to generate double-stranded DNA.

118. Dissolve HPLC grade single-stranded oligonucleotides (synthesized by General Biology System (Anhui) Co., Ltd. in powder form) in DNA binding buffer in equal molar concentration (∼0.5 mM), then mix with an equal volume of the complementary strand.

***Note:*** The design of oligonucleotides suitable for co-crystallization is extremely important. For example, the length and the ends of DNA should be considered (Patel et al., 2016). The DNA sequence should contain the core interacting base pairs 5′-CCCCTTCCCC-3′ (or 3′-GGGGAAGGGG-5′ on the complementary strand) identified by Martyn et al. (2018). We started with a 13+1 base pair double-stranded oligonucleotides (listed in the key resources table), plus a 5′-overhanging guanine or cytosine on the complementary strand. This design was lengthened by 1 bp each time until crystallization was achieved. In the end, only the 17+1 bp oligo yielded high diffraction quality crystals.

**CRITICAL:** The 17+1 bp oligo contains adenine at -194 position instead of cytosine. The rationale is that the -194 position is just outside of the known human mutations occurring at the -200 site with all of them resulting in reduced DNA binding (Martyn et al., 2018). Additionally, ITC measurement revealed nearly identical dissociation constants between wild-type (C:G pair at -194) and the mutant (A:T pair at -194) (Yang et al., 2021).

119. To anneal the DNA, incubate the mixed complementary DNA at 95°C for 5 min in a metal bath and then cool down to room temperature (∼25°C). Determine the concentration by measuring absorbance at 260 nm. Store at 4°C for up to 2 weeks.

### Crystallization of ZBTB7A protein in complex with -200 element DNA

**Timing: ∼1 year**

**Figure 4:**
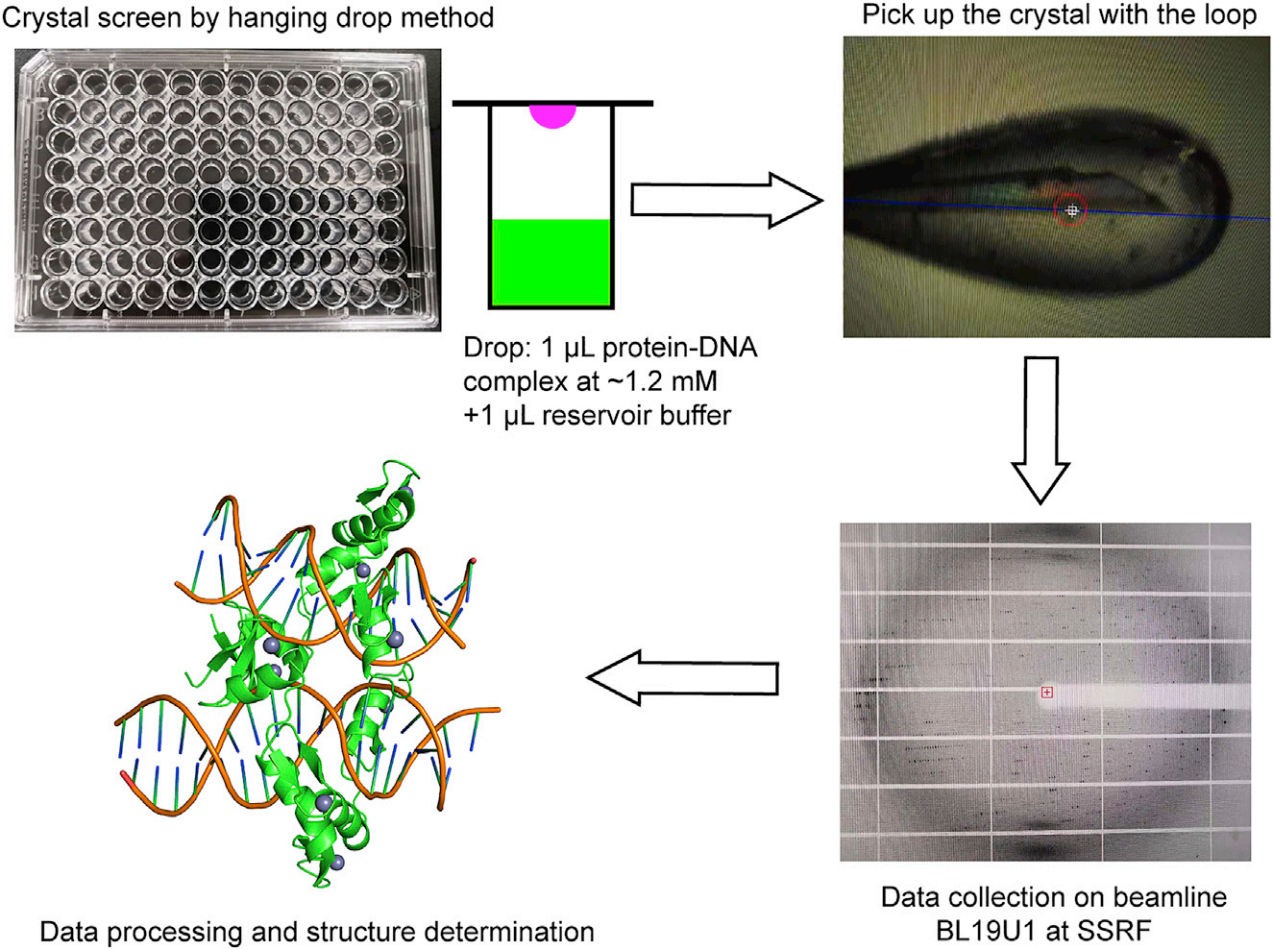
Structure determination of ZBTB7A in complex with -200 element with a C-194A mutation The crystallization process consists of crystal screening and optimization by hanging drop method, picking the crystal that is suitable for X-ray diffraction, data collection at SSRF and structure determination.

In this section crystallization and structural determination of the ZBTB7A in complex with -200 element with C-194A mutation is described (Figure 4).

120. Pool protein-containing fractions and measure the protein concentration by absorbance at 280 nm.
121. To perform protein-DNA complex formation by dialysis, mix ZF1-4 of ZBTB7A and the double-stranded DNA in a 1:1.2 molar ratio, and then dialyze the mixture against low salt DNA binding buffer at 4°C overnight (∼ 12–16 h). Transfer to a new DNA binding buffer and dialyze for another 4 h. Remove any precipitate by centrifuging at 15,871 × *g* at 4°C for 30 min. Concentrate the supernatant to ∼5 mL using an Amicon Ultra-15 Centrifugal Filter Unit (3 KDa MWCO) at a speed of 2,800 × *g* at 4°C.

***Note:*** Dialysis is a mild method used to exchange the buffer, which facilitates the protein and DNA to form a stable complex. Furthermore, overnight (∼ 12–16 h) dialysis may be performed which is also timesaving. Alternatively, protein and DNA buffer could be exchanged to low salt DNA binding buffer directly by centrifugation (repeated 3 times at least). Then, incubate protein and DNA in an appropriate molar ratio.

122. Inject the supernatant onto a HiLoad 16/600 Superdex 75 pg column pre-equilibrated with 1.5 CV low salt DNA binding buffer and run at 1 mL/min. The ZBTB7A-DNA complex elutes at ∼70 mL on this column.
123. Pool protein-DNA containing fractions and concentrate to desired concentration (∼1.2 mM in our study). Measure the absorbance at 260 nm on a spectrophotometer (the complex molar concentration is equal to absorbance at 260 nm/MW (dsDNA)), and then centrifuge at 15,871 × *g* for 20 min before crystallization trials.

***Note:*** In this step, add TCEP to a final concentration at 0.5 mM. Then incubate the sample in a 37°C water bath to promote the protein-DNA homogeneity. After that, perform the centrifugation at 15,871 × *g* at 4°C for 20 min. In our opinion, this step may have been critical to our success in obtaining well diffracted crystals.

**CRITICAL:** The concentration of the protein-DNA complex should be above 1 mM to increase the success rate of crystallization.

124. Perform crystallization screening using commercially available Index/Crystal Screen/Natrix/PEG/Ion Screen (Hampton Research), Structure Screen/Proplex Screen/The Stura Footprint Screen/Macrosol (Molecular Dimensions), The PACT Suite (QIAGEN).
125. Grow the crystals by mixing 1 μL protein-DNA complex and 1 μL reservoir buffer against 200 μL reservoir solution at 20°C via the hanging-drop vapor diffusion method.
126. Rod crystals appear in three days in 0.1 M MIB buffer (produced by mixing sodium malonate, imidazole, and boric acid in the molar ratio of 2:3:3), pH 7.0 and 25% (w/v) polyethylene glycol (PEG) 1500, which are suitable for X-ray diffraction.
127. Pick the crystals using appropriate size loops under an optical microscope. Transfer the crystals with loops and wash them with mother liquor, then in cryo-protector made of mother liquor supplemented with 25% (v/v) glycerol before being flash-frozen in liquid nitrogen.

***Note:*** The loop used to pick the crystal should be carefully selected, and the crystal should sit at the center of the loop. Additionally, the concentration of glycerol in cryo-protector should be optimized. For example, pipette 3 μL cryo-protector and insert in liquid nitrogen. If it is transparent, the concentration is appropriate. Otherwise, increase the concentration of glycerol.

128. Store crystals in liquid nitrogen and ship to the beamline BL19U1 at the Shanghai Synchrotron Radiation Facility (SSRF). The data were collected at a wavelength of 0.979 Å.
129. Process and scale the diffraction data set using the HKL-2000 (Otwinowski et al., 2003).
130. Perform crystallographic phasing by using the AutoSolve module in the PHENIX (Terwilliger et al., 2009), with the zinc atom sites being found by the SHELX C/D program (Fu et al., 2007). There are two protein-DNA molecules in an asymmetric unit calculated by Matthews Coefficient. Thus, we searched for eight zinc atoms and found seven zinc atoms by SHELX C/D, for the electron densities of ZF4 of chain H in PDB 7EYI are less ordered than that of ZF1-3.
131. Build the initial protein model using the Buccaneer program in the CCP4 suite (Collaborative Computational Project, 1994).
132. Further build the DNA model using Coot (Emsley and Cowtan, 2004), and refine using the *PHENIX.Refine* program (see troubleshooting 8).
133. Deposit final models and scaled reflection data to the PDB (our data are ID:7EYI).

### Isothermal titration calorimetry (ITC) to determine dissociation constants (*K*_D_s)

**Timing: 2 weeks**

To compare the binding affinities between ZBTB7A and WT or HPFH-associated mutant DNA, an ITC assay was chosen to measure the dissociation constants (Gontier et al., 2021). This method was also used to determine the dissociation constants between BCL11A and -115 element of γ-*globin* gene promoter to evaluate the DNA mutations occurred at G-117 and G-114 position (Yang et al., 2019).

134. Dialyze ZBTB7A protein in ITC titration buffer (20 mM Tris-HCl, pH 7.5, 150 mM or 300 mM NaCl) overnight (∼ 12–16 h), then filter the buffer for future use.
135. Concentrate the protein to the desired concentration (∼0.3–0.4 mM in our paper) as measured by absorbance at 280 nm, then centrifuge at 15,871 × *g* at 4°C for 20 min to get rid of any bubbles before ITC trials.
136. DNA annealing. For each mutant DNA sample, dissolve the DNA strands to a concentration of 100 μM, and then diluted to the desired concentration (∼20–30 μM) in the filtered ITC titration buffer.
137. Adjust the pH to the same as ITC titration buffer. Centrifuge at 15,871 × *g* at 4°C for 20 min to get rid of any bubbles before ITC trials.

***Note:*** ITC is compatible with most buffers, such as Tris and HEPES buffers. In addition, the ZBTB7A protein is very stable in Tris buffer (20 mM Tris-HCl, 150 or 300 mM NaCl, pH 7.5) as verified by SDS-PAGE, showing no aggregated or degraded bands. The C-value of the fitted curves should be considered before selecting the initial concentration of sample in the cell (Ge et al., 2020; Amano et al., 2019), because this is a critical parameter which determines the shape of the binding curves. C-value=C_*cell*_×N/*K*_D_ (C_*cell*_ is the concentration of the sample in the cell, N is stoichiometry and *K*_D_ is the dissociation constant), whereas the C_*syringe*_= 10–20×C_*cell*_. To measure *K*_D_ accurately, the C-value should be in range of 10–1000, with a value from 50-500 being ideal (Ge et al., 2020; Amano et al., 2019). We set the initial concentration of sample in the cell to 25 μM (in 150 mM NaCl) and performed the ITC assay. The C-value was 71.4 which is in the range of 50–500. This provided the rationale for the selection of the initial concentration of sample in the cell at 20–30 μM.

**CRITICAL:** Both protein and DNA samples need to be de-gassed by centrifugation before ITC trails.

138. Soak the ITC instrument in 12% Decon 90 for 1 h, then wash with the cleaning module. Conduct a water-to-water titration experiment to test the performance. In theory, the difference value of differential power (DP) should be less than 0.05. A value of less than 0.02 indicates that the ITC instrument is in good state.
139. Load 200 μL of the double-stranded oligonucleotides into sample cell, and load 40 μL of the proteins into the syringe. The titration protocol is the same for all the measurements, which is composed of a single initial injection of 1 μL protein, followed by 19 injections of 2 μL protein into DNA samples, the intervals between injections is set to 150 s, the reference 3power is 5 μcal s^−1^ and the temperature is set to 20°C (see troubleshooting 9). The reaction parameters for ITC measurements are:

**Table:** 

*Experimentalinformation*	

Syringe concentration (M)	3-4e^-4^
Cell concentration (M)	2-3e^-5^

*Instrumentsettings*	

Temperature (°C)	20
Reference Power (μcal/s)	5
Feedback	High
Stir Speed (rpm)	750
Initial Delay (s)	60

*Injectsettings*	

Total number of injections	20
Initial Volume (μL)	1
Initial Duration (s)	2
Volume (μL)	2
Duration (s)	4
Spacing (s)	150

***Note:*** The ITC titration buffer for determining dissociation constants between protein and wild type or C-194A mutant DNA contained 20 mM Tris-HCl, 150 mM NaCl, pH 7.5. The ITC titration for determining dissociation constants between protein and wild type or HPFH-associated mutant DNA contained 20 mM Tris-HCl, 300 mM NaCl, pH 7.5.

**CRITICAL:** The protein and DNA should be taken out of ice and allowed to return to experimental temperature (20°C) before ITC assays, to avoid bubbles in the process of stirring.

140. Calculate dissociation constants (*K*_D_s) by fitting the experimental data to a single-site binding model, using MicroCal PEAQ-ITC Analysis Software provided by the manufacturer. Start up the MicroCal PEAQ-ITC Analysis Software and read the ITC raw data, then adjust baseline manually if needed, fit the curves until the stoichiometry, the dissociation constant, enthalpy change, and offset remain constant and are displayed.

***Note:*** The equations that are the basis for fitting the experimental data are not described here and can be found in the MicroCal PEAQ-ITC Analysis Software User Manual.

**Figure 5:**
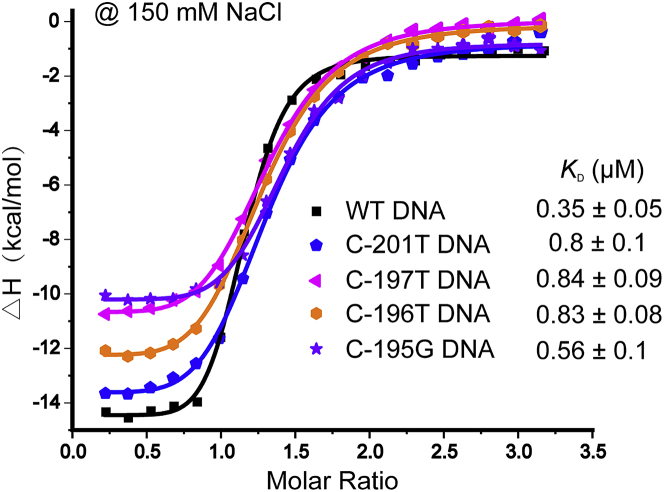
ITC evaluation of HPFH-associated mutations in 150 mM NaCl

***Note:*** ITC is a label-free method, but the experiments require more materials and high concentration. ITC allows determination of binding constants (K_D_), enthalpy (ΔH), entropy (ΔS), and the reaction stoichiometry (n), thereby an entire thermodynamic analysis of molecular binding can be obtained in an individual analysis by applying this technique. ITC is usually used to determine the binding affinities between protein-protein or protein-ligand interactions but can also be applied to measure the dissociation constants between protein-DNA interactions (Li et al., 2017; Yu et al., 2016). Recently, Yang et al. (2019) and Qiu et al. (2019) reported the complex structures of ZF proteins BCL11A and REF6 bound to γ-globin -115 element or hypo-methylated DNA motif, respectively. They further demonstrated that HPFH-associated DNA mutations substantially weaken the interaction with BCL11A, and that REF6 has a relatively low affinity for a methylated DNA motif, by ITC experiments (Yang et al., 2019; Qiu et al., 2019), which are both consistent with in vivo experiments. If target proteins are stable and able to be prepared with a high yield, ITC is a good method to detect binding affinities. Otherwise, FP assays is a good alternative to determine dissociation constants.

141. Effect of NaCl concentration. The difference of *K*_D_ values between protein and wild type or HPFH-associated mutant DNA in 150 mM NaCl were not apparent (Figure 5). Hashimoto et al. (2014) demonstrated that the WT1+KTS isoform binds most strongly to 5caC-containing DNA in 200 mM NaCl, but with a considerably lower affinity (∼50-fold decrease) in 300 mM NaCl. This suggests that *K*_D_ values are sensitive to the concentration of NaCl. We increased the concentration of NaCl in the ITC titration buffer from 150 mM to 300 mM which allowed us to determine the ITC measurements of ZBTB7A against wild type or HPFH-associated mutant DNA (Yang et al., 2021) (see troubleshooting 10).

## Expected outcomes

ZBTB7A is a well characterized transcription factor, which belongs to a sub-family of the BTB domain-containing C2H2 zinc finger proteins (Singh et al., 2021). The molecular mechanism underlying the C-terminal ZFs of ZBTB7A recognition of the γ-*globin* gene promoter remained unclear (Martyn et al., 2018). The protocol described here was used to uncover the molecular mechanism by which ZBTB7A ZFs binds to the γ-*globin* gene promoter. The outcome of the EMSA experiment illustrates that the ZBTB7A ZF binds strongly to the WT probe but that mutations at the -195 site reduce binding affinity (Figure 2).

Following the protocol described here we used ITC experiments to determine the binding affinities between the γ-*globin* gene promoter containing patient DNA mutations with ZBTB7A protein (Figure 5). Furthermore, this protocol can also be widely applied to express and purify various C2H2 ZF proteins *in vitro* after minor adaptations.

In our hands, 2 L of cell culture grown in LR medium yielded about 10 mg ZBTB7A protein. High-quality crystals of ZBTB7A in complex with -200 element DNA with C-194A mutation are expected following our protocol. We attempted to determine the structure ZBTB7A bound to the WT DNA sequence but failed to obtain diffracting crystals. There are many possibilities that could explain this, one of which is DNA length or that DNA ends selected were not suitable. Future use of additional crystallization conditions could resolve this issue.

ITC has been proven to be a powerful method to determine the dissociation constants between protein and DNA (Gontier et al., 2021). Compared with the WT -200 element DNA, patient DNA mutations indeed decreased the binding affinities with ZBTB7A protein. A similar strategy can be applied to study other ZF proteins and investigate the effects of disease-related mutations, to reveal how those mutations may affect protein function.

## Quantification and statistical analysis

The crystal structure of ZBTB7A-DNA (C-194A) was solved and refined using software listed in the key resources table. Statistics generated from X-ray crystallography data processing, refinement, and structure validation are described in (Yang et al., 2021).

ITC was performed in one trial for most samples, while the others were performed two or three times, when bubbles in sample caused a large fluctuation of the baseline. Ideally, three independent replicates are desirable. Data was processed and the *K*_D_ values were determined by curve fitting to a single-site binding model using MicroCal PEAQ-ITC Analysis Software provided by the manufacturer.

## Limitations

The protocols described here are for the characterization of ZBTB7A in complex with DNA *in vitro* in a purified system.

The EMSA experiments represent an *in vitro* model of DNA-protein interactions which may differ from the binding *in vivo* within a mammalian cell. It can be challenging to study the binding of full-length transcription factors to DNA in EMSA experiments. Often individual DNA binding domains, or combinations of DNA binding domains are used (as was used here), rather than full length proteins, to explore DNA binding in EMSA experiments.

ITC was used to measure the binding affinities between ZF1-4 of ZBTB7A and HPFH-associated DNA mutations. However, ITC experiments comparing WT ZBTB7A and ZBTB7A with mutations in residues that are responsible for recognizing DNA bases have not been performed. These experiments could be used to further experimentally explore the interaction observed in the crystal structure.

We have solved the crystal structure of ZF1-4 of ZBTB7A bound to the -200 element DNA, but how the NuRD complex is recruited by the BTB domain of ZBTB7A remains unclear (Masuda et al., 2016). Therefore, it is of great importance to explore the interaction details by solving the structure of this protein complex.

Finally, the *E. coli* expressed ZBTB7A may represent only a simplified *in vitro* interacting model. In human cells, ZBTB7A plays important roles in several fundamental biological processes by binding to different DNA elements (Maeda et al., 2005), such as hematopoiesis and oncogenic transformation (Singh et al., 2021), and it is necessary to explore functional roles in a combination of *in vivo* and *in vitro* experiments.

## Troubleshooting

### Problem 1

The polyacrylamide gel shrinks when polymerized/solidified (preparation of 6% polyacrylamide gel, step 60).

### Potential solution

Make new 10× TBE buffer.

### Problem 2

The acrylamide gel is not polymerizing, or it is taking too long to polymerize (preparation of 6% polyacrylamide gel, step 60).

### Potential solution

Make new 25% APS if it is a few weeks old. APS may also take longer to polymerize if the room is cold. If this is the case, wait longer for polymerization to occur, or move the acrylamide gel to a warmer room to polymerize more quickly.

### Problem 3

EMSA bands are too faint or do not appear on the gel after phosphor imaging (gel imaging, step 83).

### Potential solution

When performing nuclear extractions from cells, consider using a smaller volume of buffer C to concentrate the NXT. Alternatively, if a smaller volume of NXT such as 2 μL was added for each gel shift sample, increase the volume to 5 μL.

### Problem 4

EMSA bands are too strong after phosphor imaging (gel imaging, step 83).

### Potential solution

Dilute your NXT or add less NXT to your loading samples.

### Problem 5

EMSA supershift lanes appear as a smear (gel imaging, step 83).

### Potential solution

The smear appears due to too much NXT present in the sample. Reduce the amount of NXT in the supershift lane (with antibody) and in the sample without the supershift (no antibody). This may involve some trial-and-error to determine the suitable amount of protein to load for each sample.

### Problem 6

OD_600_ value of induction leads to a low expression level for ZBTB7A (expression and purification of ZBTB7A-ZF1-4, step 101).

### Potential solution

ZBTB7A protein expresses better when induced to OD_600_ 1.0–1.2 - the yield for cleaved ZBTB7A is about 5 mg/L at 16°C under these conditions. When bacteria are grown to an OD_600_ of more than 1.2 or less than 1.0 low expression or undetectable expression can result.

### Problem 7

The protein is brown after purification when expressed in LB medium (expression and purification of ZBTB7A-ZF1-4, step 107).

### Potential solution

In LB medium, the protein could chelate Fe^2+^ non-specifically. A solution is to change to LR medium instead of LB medium.

### Problem 8

ZBTB7A-DNA (WT) complex fails to be crystallized or obtain high quality diffraction data (crystallization of ZBTB7A protein in complex with -200 element DNA, step 132).

### Potential solution

Firstly, check the purity of the protein purification by SDS-PAGE. Secondly, change the length and ends of the interacting DNA sequence. Thirdly, increase the protein-DNA complex concentration to above 1 mM. Fourthly, a crystallization screen with matrices of the known conditions should be performed for each new construct, followed by new crystallization screens and various temperature (10°C or 20°C) if needed. Lastly, slightly modify the DNA sequence outside of core interacting sequence. Another alternative is post-crystallization treatment of crystals.

### Problem 9

The reaction heat (the heat change that occurs when two molecules interact, which is a common term used in ITC assays) of ITC is too low to measure (isothermal titration calorimetry (ITC) to determine dissociation constants (KDs), step 139).

### Potential solution

Increase the protein and DNA concentration. The protein concentration should be ten times higher than the DNA concentration.

### Problem 10

The ITC assays for some DNA mutants in 150 mM NaCl does not show desired results ((isothermal titration calorimetry (ITC) to determine dissociation constants (KDs), step 141).

### Potential solution

Typically, the ITC assays results should be consistent with data from crystallization experiments. For example, the ITC assays should be performed in crystallization buffer (150 mM NaCl). However, in our study, the difference of *K*_D_ values between protein and wild type or HPFH-associated mutant DNA in 150 mM NaCl were not apparent, which is not consistent with what we observed from crystal structure. This may be caused by strong hydrogen bonding contributed by base-specific interactions and phosphate bone interactions. Check the DNA sequence and make sure it is appropriate, and then increase the salt concentration to 300 mM NaCl for further ITC titration. This adjustment led to our successful ITC results.

## Resource availability

### Lead contact

Further information and requests for resources and reagents should be directed to and will be fulfilled by the lead contact, Kate Quinlan (kate.quinlan@unsw.edu.au).

### Materials availability

ZBTB7A ZF plasmids generated in this study are available upon request.

## Data Availability

The atomic coordinates and structural factors for the crystal structure of ZBTB7A (382–506) bound to -200 element with C-194A mutation in this paper has been deposited to the Protein Data Bank (PDB) under accession number 7EYI.
