# Supplementary material for: Protocols for protein-DNA binding analysis of a zinc finger transcription factor bound to its cognate promoter
Source: STAR Protoc. 2022 Jul 31;3(3):101598. doi: 10.1016/j.xpro.2022.101598 (PMC9344028; doi:10.1016/j.xpro.2022.101598)
Supplement: Document S1. Table S1 [file mmc1.pdf]

Table S1

**Oligonucleotides**		
-200γ WT-forward<br>TGGGGGCCCCTTCCCCACACTAT (for EMSA)	Martyn *et al*. 2018	N/A
-200γ WT-reverse<br>ATAGTGTGGGGAAGGGGCCCCCA (for EMSA)	Martyn *et al*. 2018	N/A
-195 C>G-forward<br>TGGGGGCCCCTTCCGCACACTAT (for EMSA)	Martyn *et al*. 2018	N/A
-195 C>G-reverse<br>ATAGTGTGCGGAAGGGGCCCCCA (for EMSA)	Martyn *et al*. 2018	N/A
-195 C>T-forward<br>TGGGGGCCCCTTCCTCACACTAT (for EMSA)	Yang *et al.* 2021	N/A
-195 C>T-reverse<br>ATAGTGTGAGGAAGGGGCCCCCA (for EMSA)	Yang *et al.* 2021	N/A
-195 C>A-forward<br>TGGGGGCCCCTTCCACACACTAT (for EMSA)	Yang *et al.* 2021	N/A
-195 C>A-reverse<br>ATAGTGTGTGGAAGGGGCCCCCA (for EMSA)	Yang *et al.* 2021	N/A
ZBTB7A primer-forward<br>5'-GGAATTCCATATGCAGAAGTGCCCCA<br>TCTGCGAGAAG-3' (for PCR)	This paper	N/A
ZBTB7A primer-reverse<br>5'-CCGCTCGAGTTACCACCCGCCCCGGA<br>CGCGGGGCTTGC-3' (for PCR)	This paper	N/A
WT DNA-forward<br>5'-GGCCCCTTCCCCA-3' (for ITC)	This paper	N/A
WT DNA-reverse<br>5'-TGGGGAAGGGGCC-3' (for ITC)	This paper	N/A
G-204A DNA-forward<br>5'-GACCCCTTCCCCA-3' (for ITC)	This paper	N/A
G-204A DNA-reverse<br>5'-TGGGGAAGGGGTC-3' (for ITC)	This paper	N/A
C-203G DNA-forward<br>5'-GGGCCCTTCCCCA-3' (for ITC)	This paper	N/A
C-203G DNA-reverse<br>5'-TGGGGAAGGGCCC-3' (for ITC)	This paper	N/A
C-202G DNA-forward<br>5'-GGCGCCTTCCCCA-3' (for ITC)	This paper	N/A
C-202G DNA-reverse<br>5'-TGGGGAAGGCGCC-3' (for ITC)	This paper	N/A
C-202T DNA-forward<br>5'-GGCTCCTTCCCCA-3' (for ITC)	This paper	N/A
C-202T DNA-reverse<br>5'-TGGGGAAGGAGCC-3' (for ITC)	This paper	N/A
C-201T DNA-forward<br>5'-GGCCTCTTCCCCA-3' (for ITC)	This paper	N/A
C-201T DNA-reverse<br>5'-TGGGGAAGAGGCC-3' (for ITC)	This paper	N/A
T-198C DNA-forward	This paper	N/A

5’-GGCCCCTCCCCCA-3’ (for ITC)		
T-198C DNA-reverse<br>5’-TGGGGGAGGGGCC-3’ (for ITC)	This paper	N/A
C-197T DNA-forward<br>5’-GGCCCCTTTCCCA-3’ (for ITC)	This paper	N/A
C-197T DNA-reverse<br>5’-TGGGAAAGGGGCC-3’ (for ITC)	This paper	N/A
C-196T DNA-forward<br>5’-GGCCCCTTCTCCA-3’ (for ITC)	This paper	N/A
C-196T DNA-reverse<br>5’-TGGAGAAGGGGCC-3’ (for ITC)	This paper	N/A
C-195G DNA-forward<br>5’-GGCCCCTTCCGCA-3’ (for ITC)	This paper	N/A
C-195G DNA-reverse<br>5’-TGCGGAAGGGGCC-3’ (for ITC)	This paper	N/A
13+1 bp DNA-forward<br>5’-GCCCCTTCCCCACA-3’ (for crystallization)	This paper	N/A
13+1 bp DNA-reverse<br>5’-CTGTGGGGAAGGGG-3’ (for crystallization)	This paper	N/A
14+1 bp DNA-forward<br>5’-TGGGCCCCTTCCCCA-3’ (for crystallization)	This paper	N/A
14+1 bp DNA-reverse<br>5’-ATGGGGAAGGGGCCC-3’ (for crystallization)	This paper	N/A
15+1 bp DNA-forward<br>5’-AGGGCCCCTTCCCCAC-3’ (for crystallization)	This paper	N/A
15+1 bp DNA-reverse<br>5’-TGTGGGGAAGGGGCCC -3’ (for crystallization)	This paper	N/A
16 bp DNA-forward<br>5’-GGGCCCCTTCCCCACA-3’ (for crystallization)	This paper	N/A
16 bp DNA-reverse<br>5’-TGTGGGGAAGGGGCCC -3’ (for crystallization)	This paper	N/A
16+1 bp DNA-forward<br>5’-TGGGCCCCTTCCCCACA-3’ (for crystallization)	This paper	N/A
16+1 bp DNA-reverse<br>5’-ATGTGGGGAAGGGGCCC-3’ (for crystallization)	This paper	N/A
17+1 bp DNA-forward<br>5’-ATAGGGCCCCTTCCCAAC-3’ (for crystallization)	This paper	N/A
17+1 bp DNA-reverse<br>5’-TGTTGGGAAGGGGCCCTA-3’ (for crystallization)	This paper	N/A
